# Primary pulmonary mucosa-associated lymphoid tissue lymphoma: A case report

**DOI:** 10.1016/j.radcr.2022.09.038

**Published:** 2022-10-08

**Authors:** Mohammad Reza Hosseini Siyanaki, Elham Askari, Sara Haseli, Nazanin Sadraei

**Affiliations:** a Shahed University Faculty of Medical Sciences, Tehran, Iran; b Chronic Respiratory Diseases Research Center, National Research Institute of Tuberculosis and Lung Diseases (NRITLD), Shahid Beheshti University of Medical Sciences, Tehran, Iran; c Medical Imaging Research Center, Shiraz University of Medical Sciences, Shiraz 7193613311, Iran

**Keywords:** Lymphoma, Pulmonary, Primary, MalToma, CT scan, Dyspnea

## Abstract

Primary pulmonary lymphoma (PPL) is a rare entity with the most common presentation as mediastinal lymphadenopathy. The most common form of PPL is Mucosa-Associated Lymphoid Tissue Lymphoma (MALToma) which is an extranodal B-cell lymphoma originating from the mucosal layers involving different organs such as the gastrointestinal tract as well as the lung. Herein, we present a case of a 51-year-old woman with progressive dyspnea for 6 months and no prior medical history. The computed tomography (CT scan) revealed bilateral multifocal consolidation and ground-glass opacities as well as interlobular septal thickening. Bronchoscopy was normal and CT-guided biopsy of lung consolidations was conclusive of MALToma. Complete extrapulmonary evaluations inducing bone marrow aspiration were unremarkable. The primary pulmonary MALToma is an extremely rare entity that presents with non-specific symptoms and a wide variety of CT findings such as mediastinal, hilar lymphadenopathy, and single or multiple lung nodules ranging from 2 to 8 cm. the disease has a favorable prognosis, so prompt diagnosis is essential.

## Introduction

Primary pulmonary lymphoma is a very rare neoplasm that accounts for 0.4% of all lymphomas [1]. The most common presentation is mediastinal lymphadenopathy and to a lesser extent parenchymal involvement with 38% in Hodgkin lymphoma (HL) and 24% in non-Hodgkin lymphoma (NHL) [2]. Mucosa-associated lymphoid tissue (MALT) lymphoma is an extranodal B-cell lymphoma originating from the mucosal layers with the most common location of MALT lymphoma being the gastrointestinal system, thyroid, and salivary glands; however, primary pulmonary MALT lymphoma is extremely rare [3]. It may happen as a result of chronic inflammation either in the setting of infectious or autoimmune conditions. Meanwhile, 16% of pulmonary MALT lymphoma patients had a positive history of autoimmune diseases [4,5]. Here, we present a case of a 51-year-old woman with progressive dyspnea and a final diagnosis of primary pulmonary MALT lymphoma.

## Case report

In July 2022, a 51-year-old woman with the complaint of progressive dyspnea and dry cough for 6 months was referred to Masih Daneshvari hospital, Tehran, Iran. She had no prior medical conditions and did not smoke. On admission, the temperature was 37°C, with a pulse rate of 75 bpm/min, blood pressure of 120/85 mm Hg, respiration rate 20/min, and oxygen saturation of 93% in room air. Bilateral scattered crackles were the only abnormal physical examination.

**Fig. 1:**
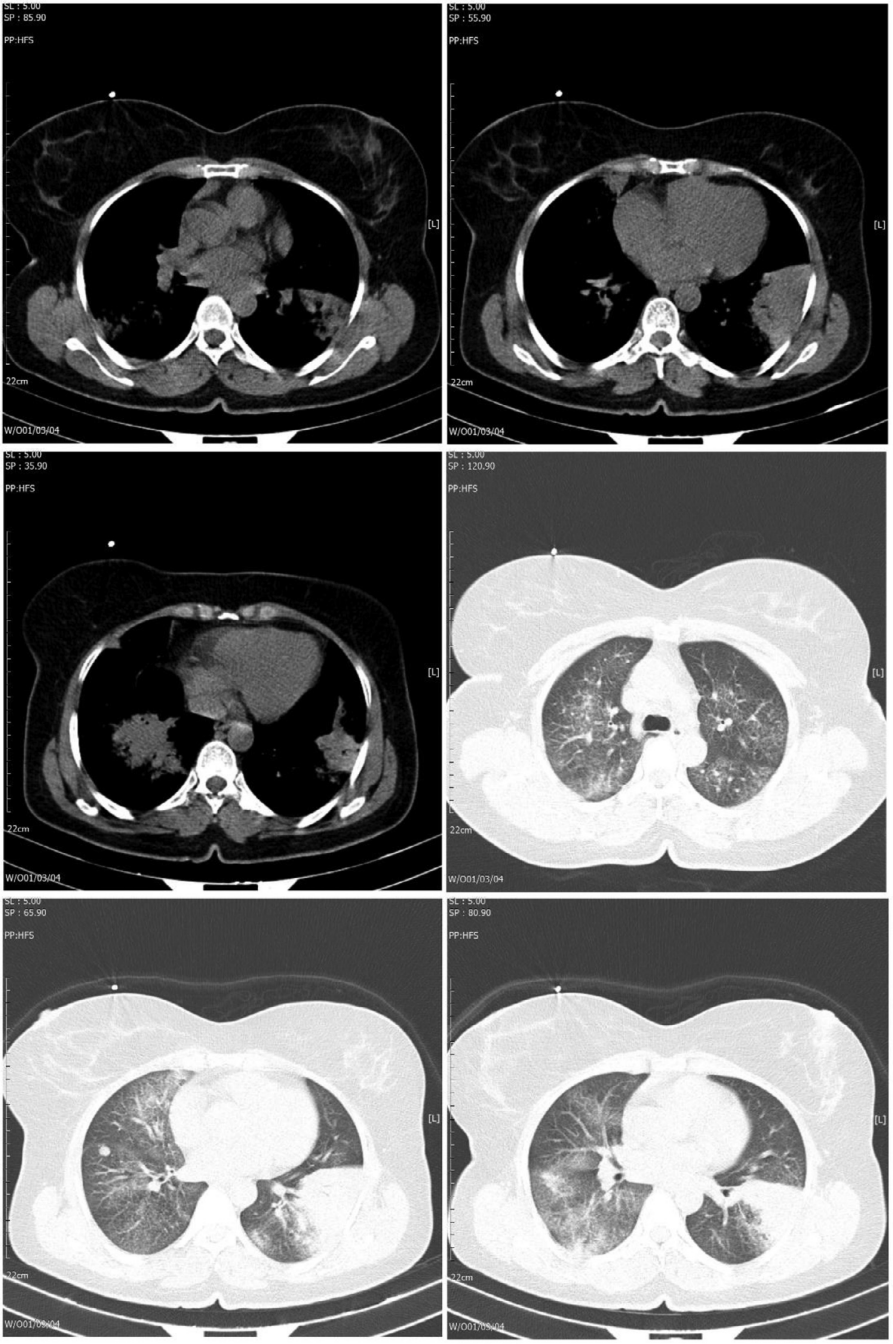
Non-contrast computed tomography (A, B, C) mediastinal window, (C, D, and E) lung window, show interlobular septal thickening and ground-glass opacities (GGO) bilaterally. Left lower lobe consolidation and GGOs with scattered interlobular septal thickening.

**Fig. 2:**
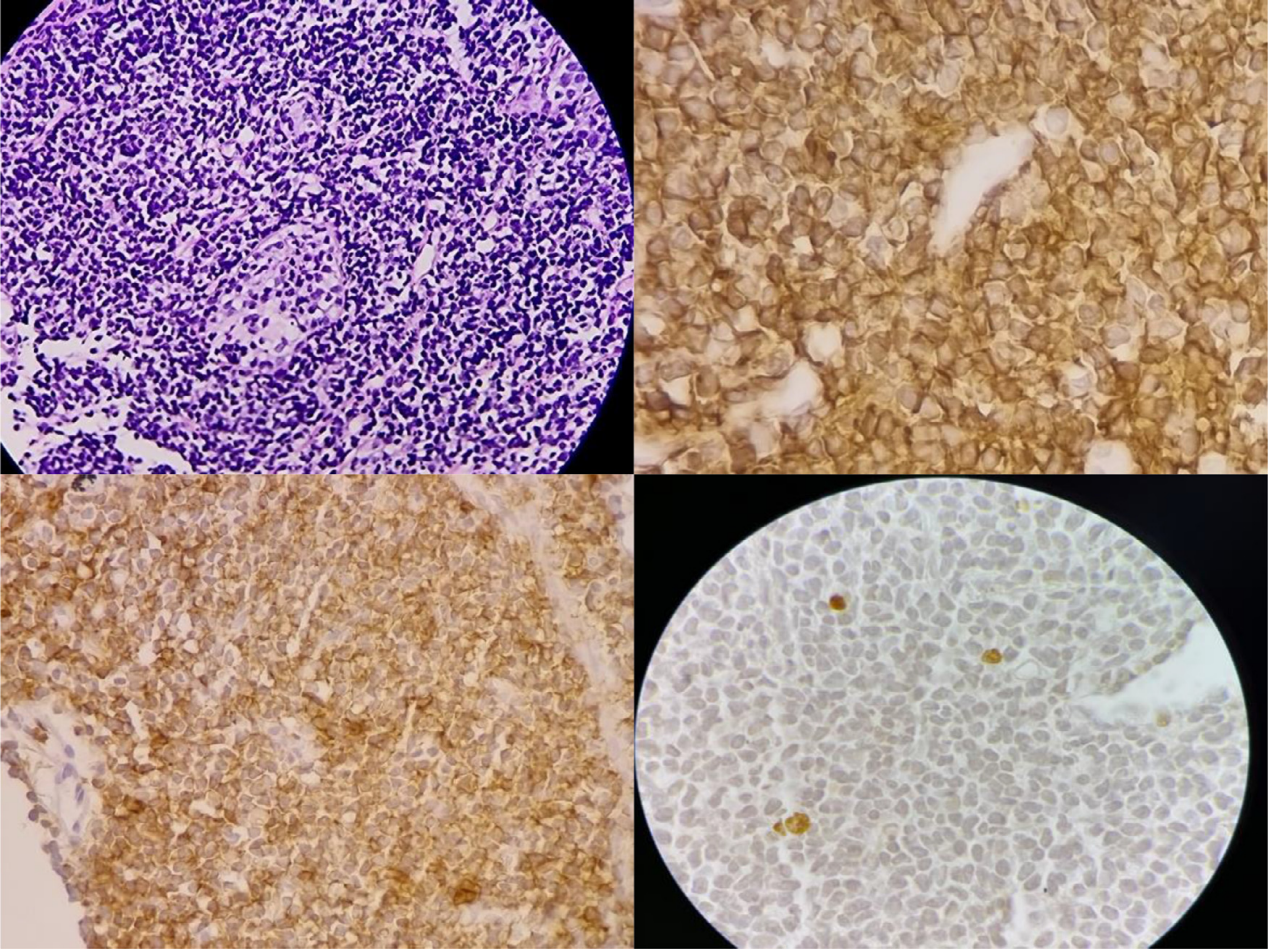
Morphology and immunohistochemistry. (A) Hematoxylin and Eosin (H&E) stained sections reveal sheets of small- to medium-sized lymphoid population with pale cytoplasm. (B) shows CD20 is positive, and lymphocytes B are abundant. (C) In immunohistochemistry, this image shows a lymphoepithelial lesion, CD43. (D) This image shows the positive rate of Ki67 was <5%.

Non-contrast CT of the chest showed bilateral multifocal consolidation and ground-glass opacities as well as interlobular septal thickening (Fig. 1). Bronchoscopy was performed; it showed normal airways with no evidence of endoluminal lesion, and the bronchoalveolar lavage was negative. CT-guided biopsy of the lung consolidations was performed. Hematoxylin and Eosin (H&E) stained sections revealed sheets of the small- to a medium-sized lymphoid population with pale cytoplasm (Fig. 2A).

The lung parenchyma, including bronchi mucosa and peribronchial interstitium, was obliterated by the lymphocytic cells. In addition, apparent lymphoepithelial lesions were also seen. The neoplastic cells showed positive immunoreaction by CD20 (Fig. 2B), CD43 (Fig. 2C), CD79a, CD19, CD21, and the positive rate of Ki67 was < 5% (Fig. 2D), but they were negative for CD10, CD5, CD23, CyclinD1.

Complete extrapulmonary evaluations were implicated, and abdominal CT and neck ultrasonography showed no evidence of lymphadenopathy or hepatosplenomegaly. Furthermore, a bone marrow biopsy was performed, which was unremarkable. These findings support a diagnosis of low-grade B-cell NHL that was mostly consistent with marginal zone lymphoma; therefore, primary MALT lymphoma was diagnosed. The patient was referred to an oncologist for proper treatment.

## Discussion

Pulmonary NHL is a rare entity occurring 1:313,000 annually with <1% of all NHLs, in which MALT lymphoma is seen in more than two-thirds of cases [6,7]. The first MALT lymphoma arising from the gastrointestinal tract was reported in 1983 by Isaacson and Wright [8]. It occurs when the mucosal B-cells proliferate uncontrollably. There is some evidence of higher prevalence among patients with chronic immune system activation which may indicate the possibility of DNA errors caused by continuous replication resulting in cancer cell proliferation [9]. MALT lymphomas can exhibit a variety of cytogenetic abnormalities and the most common genetic abnormality is t(11;18) [10].

Lung B-cell lymphoma is the most common type of primary pulmonary lymphoma (70%-80%) [11]. It may affect immunocompetent patients as well as immunocompromised ones. MALT lymphoma has various clinical presentations as a result of different sites of involvement [12]. Most patients present with a localized form of the disease with a favorable prognosis; about one-third of the cases present with nonspecific symptoms such as dyspnea, cough, shortness of breath, chest pain, weight loss, exertional dyspnea, fever, fatigue, night sweats, and hemoptysis [13]. These non-specific symptoms may cause misdiagnosis as viral or bacterial pneumonia, cancer, sarcoidosis, or tuberculosis [14,15]. It is not related to smoking or occupational exposure, and the median age at the time of diagnosis is about 50 [11,16]. It is a challenging diagnosis due to its indolent nature, and non-specific clinical and radiological findings [17].

Some laboratory findings such as anemia, thrombocytopenia, and an elevated level of LDH are often found. In addition, monoclonal immunoglobulin (Ig) and C-reactive protein levels are usually elevated [18]. Computer tomography (CT) is often used when the patient presents with respiratory symptoms, for example, cough and shortness of breath [19].

MALT-lymphoma has heterogeneous imaging findings, most of which are nonspecific. However, some features such as hilar or mediastinal lymphadenopathy may contribute to the proper diagnosis. Single or multiple lung nodules ranging from 2 cm to 8 cm with or without air bronchogram are commonly found in lower lobes which account for up to 70% of cases [20,21]. Cavitation is not an uncommon finding, especially in larger nodules. Pleural effusion is reported in about 10% of the patients [22,23].

In most cases, positron emission tomography (PET)-CT shows increased fluorine-deoxyglucose (FDG) uptake correlating with tumor size; therefore, it can be utilized in both initial diagnosis and follow-up [24].

Bronchoscopy is usually unremarkable although bronchial edema, inflammation, stenosis, or narrowing caused by external compression can be observed [11]. Bronchoalveolar lavage is valuable for excluding the underlying infectious process [25,26].

Transbronchial biopsy has high sensitivity and specificity [27] in primary pulmonary lymphoma by demonstrating lymphocytic alveolitis [26]. In a study by Borie et al., in 84% of MALT lymphoma cases, more than 15% of them had lymphocytic alveolitis and B-cells accounted for more than 10% of alveolar lymphocytes [28]. MALT lymphoma is also characterized by a lymphoepithelial proliferation invading the bronchial epithelium [22]. Primary pulmonary MALT lymphomas have a favorable 5-year survival rate of 90% and 10-year of 70%, respectively [29].

The most common treatment option for localized or peripheral masses is surgical resection. It is usually combined with radiotherapy. Rituximab is indicated for the treatment of patients with unresectable or widespread dissemination of tumors [30,31]. However, further research is warranted to determine the most effective treatment.

## Conclusion

Primary pulmonary MALT lymphoma is a rare lung NHL with a favorable prognosis. It usually has nonspecific clinical manifestations and radiological findings. Detailed histopathological evaluation is vital for proper diagnosis. Awareness of this entity is essential for a physician to correctly diagnose the disease in the proper clinical setting.

## Ethics approval

This observational retrospective study was approved by Shahid Beheshti University of Medical Science institutional ethics committee.

## Patient consent

Informed consent was obtained from the patient.
